# Detrusor Arreflexia as an End Stage of Neurogenic Bladder in HAM/TSP?

**DOI:** 10.1155/2011/289389

**Published:** 2011-04-13

**Authors:** Matheus Tannus, Davi Tanajura, Michael A. Sundberg, Paulo Oliveira, Neviton Castro, André Muniz Santos

**Affiliations:** Serviço de Imunologia, Hospital Universitário Proffessor Edgard Santos, 5 Andar, Rua João das Botas S/N, Canela, Salvador CEP 40110-160, BA, Brazil

## Abstract

The HTLV-1 virus is a known agent involved in the development of HAM/TSP. Past studies have typically observed patients with autonomic dysfunction consisting of detrusor overactivity and detrusor-sphincter dyssynergia, with the occasional observation of underactive detrusor or detrusor arreflexia. However, studies have not yet evaluated the progression of neurogenic bladder over time. In this paper, we describe a HAM/TSP patient with the initial development of overactive detrusor, and subsequent development of detrusor arreflexia. Given a paucity of studies characterizing the effects of HTLV-1 on the autonomic nervous system, particularly aspects controlling continence, this patient's clinical course may represent one type of end point for patients with HAM/TSP and neurogenic bladder. Further cohort or case-series studies, with particular emphasis on the progression of neurogenic bladder, are needed to evaluate the significance of this described case in relation to typical disease progression patterns.

## 1. Introduction

The Human T-Lymphotropic Virus Type 1 (HTLV-1) is an RNA virus and a known agent involved in the development of HTLV-1-associated myelopathy/tropical spastic paraparesis (HAM/TSP) [1]. Speculation exists as to the current prevalence of HTLV-1 infection [2], but the most widely quoted estimate in the literature continues to be 10–20 million people worldwide [3]. Of those infected with HTLV-1, it is estimated that about four percent develop HAM/TSP [4]. The infection is endemic in tropical and subtropical areas, with higher prevalence in Central and South America, the south of Japan, and Africa [5].

HAM/TSP is a chronic myelopathy with a clinical presentation of spastic paraparesis, including pyramidal effects such as hyperreflexia and Babinski sign. Autonomic dysfunction is also observed, as well as occasional mild sensation abnormalities and/or pain [6]. Overactive bladder (OAB) is the most common autonomic presentation in patients with HAM/TSP but can also occur as an isolated form in patients with HTLV-1 infection and without paraparesis [5, 7]. Urodynamic studies in individuals with HAM/TSP alongside symptoms of frequency and difficulty voiding typically reveal detrusor overactivity (DO) and detrusor-sphincter dyssynergia (DSD) compatible with a myelopathy picture [8]. Studies have also found individuals with underactive or detrusor arreflexia (DA) and resulting overflow incontinence, though this is less commonly observed [9, 10].

In this paper, our objective is to describe a HAM/TSP patient with initial OAB due to DO, and subsequent development of DA and overflow incontinence.

## 2. Case Presentation

The patient is a 70-year-old female with HTLV-I infection and a five-year diagnosis of HAM/TSP. Two years ago, the patient developed OAB initially characterized by urgency with incontinence and nocturia. However, over the past six months, the patient ceased to have urgency and began requiring regular self-catheterization for voiding. The patient does not have a history of diabetes, hypertension, syphilis, or HIV.

On a routine outpatient neurologic exam, the patient was alert and oriented. Proximal asymmetric paraparesis grade 4 (Medical Research Council [11]) was noted on flexion of the leg at the right hip, and grade 4+ was noted on flexion of the leg at the left hip. Grade 2 spasticity (Ashworth Scale [12]) was observed on passive movement of the legs. Global hyperreflexia, grade 3 (Campbell Score [13]), and bilateral Babinski sign were also noted. Sensation was normal, and there was no alteration in coordination nor in equilibrium. The patient was classified according to the Kurtzke Expanded Disability Status Scale (EDSS) [14] and the Osame Disability Motor Scale (ODMS) [15], with scores of 4 and 5, respectively.

**Figure 1:**
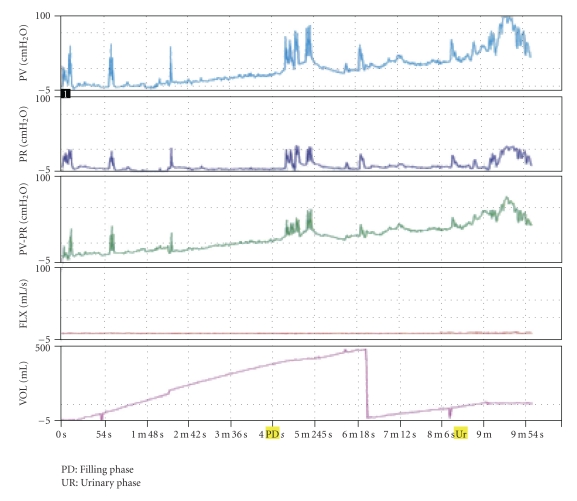
Urodynamic study performed on patient (2008) showing detrusor hyperreflexia prior to voiding. PV: vesicular pressure, PR: abdominal pressure, PV-PR: detrusor pressure, Vol: volume in bladder.

**Figure 2:**
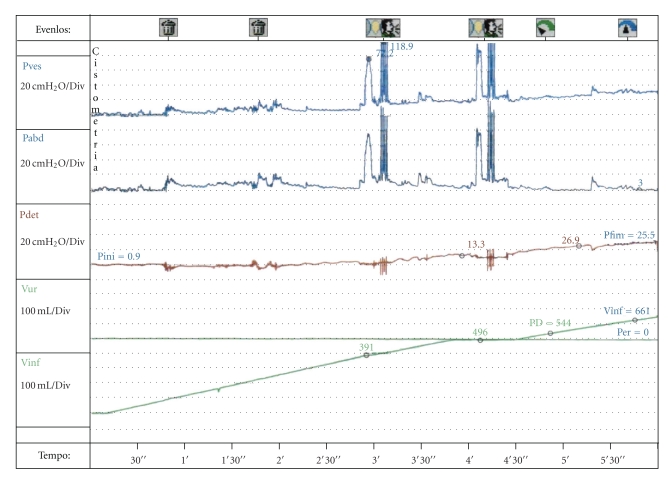
Urodynamic study performed on patient (2010) displaying detrusor arreflexia. Pves: vesicular pressure, Pabd: abdominal pressure, Pdet: detrusor pressure, Vur: urine volume, Vinf: infusion volume.

Urodynamic studies were conducted on this patient in both 2008 and 2010. In these studies, a 12 Fr trilumen catheter was transurethrally inserted into the bladder to measure vesicular pressure, while abdominal pressure was measured by intrarectal balloon catheter. Detrusor pressure was obtained by subtracting abdominal pressure from bladder pressure. In 2008, the studies revealed OAB with DSD and OD (Figure 1). However, studies in 2010 revealed DA and an inability to void during pressure-flow studies (Figure 2), consistent with the patient's loss of urgency and continued incontinence.

**Figure 3:**
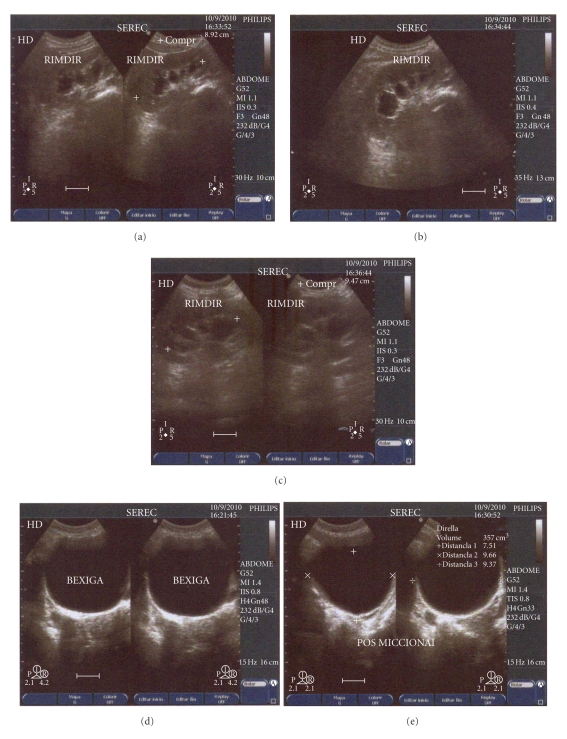
Urinary sonogram performed on patient (2010) revealing grade II hydronephrosis consistent with noncontractile bladder.

A urinary sonogram performed in 2010 notes a postvoid residual volume of 357 cm^3^ and bilateral, grade II hydronephrosis with communicative hypoechoic areas observed in both kidneys (Figure 3); this is consistent with the effects of overflow incontinence due to noncontractile bladder. At this time, the right kidney measured 8.92 cm in greatest diameter, while the left kidney measured 9.47 cm. The patient's most recent routine urine analysis and cultures in 2010 were unremarkable, with creatinine and blood urea nitrogen (BUN) within normal range and indicating preserved renal function.

Initially, upon development of OD, the patient was treated with anticholinergics. However, prevention of residual urine now requires regular catheterization. The patient was educated to perform self-catheterization and tolerates the procedure well.

## 3. Discussion

OAB and OD, with or without DSD, are a common feature in patients with HTLV-1, especially those with HAM/TSP. In a urodynamic evaluation of HTLV-1-infected patients with and without HAM/TSP, de Castro et al. [8] described OD in 37% of HTLV-1 patients without HAM/TSP and in 46.9% of patients with a diagnosis of HAM/TSP. Alongside OD, DSD was described in 11% and 34% of the patients, respectively. However, DA was not described in any patients in this study. Likewise, similar studies by Lima et al. [16], Imamura et al. [10], and Yamashita and Kumazawa [17] observed OD ranging from 60–96% among patients with HAM/TSP. Lima and Yamashita also noted DSD in 35% and 65% of HAM/TSP patients, respectively.

Few studies have described patients with DA. In Yamashita's study of 26 HAM/TSP patients, only one patient was observed to have DA on urodynamic evaluation. In a similar fashion, in a study of 25 HAM/TSP patients, Imamura found DA in four (16%) patients and hydronephrosis in two (8%). And, though both OD and DA have been observed in past studies of patients with HAM/TSP, these studies were not conducted with the purpose of providing information regarding the temporal progression of autonomic dysfunction in HAM/TSP. 

This paper describes a patient with known HAM/TSP who first developed OD, with progression to DA after two years. As no cohort studies have yet described the progression of neurogenic bladder in HAM/TSP patients over time, it is unknown whether this patient's manifestations correspond to the natural history of bladder dysfunction in HAM/TSP or to an alternative and less-common form of progression. A progression from OD to underactive detrusor, with the eventual development of DA, may correspond with an initial lesion in the spinal cord nuclei and tracts and a subsequent neuronal secondary degeneration of bladder innervation. 

Interestingly, Komine et al. [9] described two patients similar to our case with underactive detrusor, three and six years after the diagnosis of HAM/TSP. Yet, in a previous study [18] by this group, 16 patients all with OD and most with DSD had an average duration of HAM/TSP of 12.7 years. Such information suggests the possibility of a reverse progression of autonomic dysfunction from that suggested above, with OD occurring as a later manifestation, or possibly that the development of DA is an altered course of neurologic pathology in some patients and occurs for yet unknown reasons. Cohorts with repeat urodynamic studies are needed to evaluate the progression of neurological symptoms in more patients and to determine whether a temporal progression from OD to DA is an alternative outcome in HAM/TSP patients.

From a clinical perspective, as there is a large range in urinary symptoms among patients with HTLV-1 infection (particularly those with HAM/TSP), it is important to regularly re-evaluate patients for changes in bladder function and provide specific treatment options.
